# A longitudinal study of child and adolescent psychopathology in conditions of the war in Ukraine

**DOI:** 10.1192/j.eurpsy.2024.199

**Published:** 2024-08-27

**Authors:** T. Skrypnyk, I. Martsenkovsky, I. Martsenkovska

**Affiliations:** Department of Mental Disorders of Children and Adolescents, SI «Institute of Psychiatry, Forensic Psychiatric Examination and Monitoring of Drugs of the Ministry of Health of Ukraine», Kyiv, Ukraine

## Abstract

**Introduction:**

According to UNICEF, 2 million children have left the country since the beginning of the war. 2.5 million Ukrainian children are internally displaced persons. Minors often become victims or witnesses of violence.

The events of 2022-2023 are the largest military conflict in the world since World War II. The impact on the mental health of the population is characterized by the variety and mass of traumatizing factors.

Mental trauma causes PTSD, depressive disorders (DD), anxiety disorders (AD), behavioral disorders (CD), attention deficit hyperactivity disorder (ADHD).

**Objectives:**

The aim of the study was to determine the prevalence of PTSD and its comorbidities at different stages of experiencing a traumatic experience.

**Methods:**

785 teen`s displaced from the zone of military operations, occupied territories were surveyed. Examinations included: K-SADS-PL, PSC-17, SCARED, CATS. 260 teen`s were examined during - 6, 400 – 6–12 months after traumatization.

**Results:**

After 6 months of trauma, PTSD was diagnosed in 9.8%, ADHD – 10.2%, DD-22.3%, AD-30.8%, CD – 15.4%, 28.8%; examined 6 to 12 months after the injury, respectively: 21.9%, 12.6, 33.3%, 11.5%, 18.0%.

**Conclusions:**

In war-affected children, PTSD is a risk factor for the subsequent development of comorbid depression, anxiety, conduct disorders, and ADHD. Female sex, secondary traumatization after displacement increase the risk of developing depression, signs of pervasive development and ADHD - the risk of destructive and self-injurious behavior. The prevalence of PTSD, DD, ADHD increases within 6-12 months after the trauma, the sensitivity of children with PTSD to secondary traumatic events increases.

**Disclosure of Interest:**

None Declared

